# Variability of Stepping during a Virtual Reality Paradigm in Parkinson’s Disease Patients with and without Freezing of Gait

**DOI:** 10.1371/journal.pone.0066718

**Published:** 2013-06-21

**Authors:** Moran Gilat, James M. Shine, Samuel J. Bolitho, Elie Matar, Yvo P. T. Kamsma, Sharon L. Naismith, Simon J. G. Lewis

**Affiliations:** 1 Brain and Mind Research Institute, The University of Sydney, Sydney, NSW, Australia; 2 Center for Human Movement Sciences, University of Groningen, Groningen, The Netherlands; Oregon Health & Science University, United States of America

## Abstract

**Background:**

Freezing of gait is a common and debilitating symptom affecting many patients with advanced Parkinson’s disease. Although the pathophysiology of freezing of gait is not fully understood, a number of observations regarding the pattern of gait in patients with this symptom have been made. Increased ‘Stride Time Variability’ has been one of the most robust of these features. In this study we sought to identify whether patients with freezing of gait demonstrated similar fluctuations in their stepping rhythm whilst performing a seated virtual reality gait task that has recently been used to demonstrate the neural correlate of the freezing phenomenon.

**Methods:**

Seventeen patients with freezing and eleven non-freezers performed the virtual reality task twice, once whilst ‘On’ their regular Parkinsonian medication and once in their practically defined ‘Off’ state.

**Results:**

All patients displayed greater step time variability during their ‘Off’ state assessment compared to when medicated. Additionally, in the ‘Off’ state, patients with freezing of gait had greater step time variability compared to non-freezers. The five steps leading up to a freezing episode in the virtual reality environment showed a significant increase in step time variability although the final three steps preceding the freeze were not characterized by a progressive shortening of latency.

**Conclusions:**

The results of this study suggest that characteristic features of gait disturbance observed in patients with freezing of gait can also be demonstrated with a virtual reality paradigm. These findings suggest that virtual reality may offer the potential to further explore the freezing phenomenon in Parkinson’s disease.

## Introduction

Freezing of gait (FOG) is a debilitating symptom that affects over 50% of patients with advanced Parkinson’s disease (PD) [1]. This paroxysmal phenomenon is defined by the absence or marked reduction in forward progression of the feet despite the intention to walk [2]. This symptom impairs mobility, may lead to falls and is associated with nursing home placement and a decreased quality of life [3]–[5].

Previous studies have identified that those PD patients who suffer from FOG display specific changes in gait, most notably having a generally increased stride time variability [6]–[8]. The stride time variability measures stride-to-stride fluctuations in gait and is derived from the coefficient of variability (CV), where CV = 100*Standard Deviation/Mean. It is believed to reflect the ability to produce rhythmical stepping movements without the need for conscious control [9]. A high stride time variability value is predictive of worse postural instability and an increased risk of falls [10]. Compared to non-freezers, patients with FOG have greater stride time variability, even when discounting discrete episodes of FOG during clinical assessment [8]. In addition, the stride time variability is partially ameliorated by dopaminergic medication in PD with increased variability being reported in the ‘Off’ state [7]–[10].

Previously, Moreau *et al* [11] have described an increased stride length variability in the five steps preceding a freezing episode. It is possible that a proportion of this variability may be related to the reported ‘sequence effect’, where there is a reduction in stride length in the three steps directly preceding an episode of FOG episode [12], [13].

To better understand the freezing phenomenon, our group has developed a Virtual Reality (VR) task in which subjects navigate a realistic three-dimensional environment presented in the first person by using foot pedals to control their ‘walking’. Initial work with this paradigm demonstrated that a number of measures, including the longest spontaneous pause in stepping were significantly correlated with self-reported symptoms of FOG [14]. Subsequently, the amount of freezing recorded by this VR task has been correlated with actual FOG during timed up-and-go (TUG) tasks [15]. Furthermore, the VR paradigm has been utilized in combination with functional Magnetic Resonance Imaging (fMRI) to demonstrate the neural correlates underlying freezing behaviour [16], [17] and the effects of dual-tasking in patients with FOG [18].

Although the VR task does not directly allow for the calculation of true stride parameters, it does record step time variability, which is an objective measure of the dynamic rhythm of motor activity in the lower limbs. We hypothesized that the VR task would reproduce the key features of gait variability in PD patients. Specifically, we predicted: *i)* that step time variability (STV^VR^) would be greater in all patients during their ‘Off’ state; *ii)* that patients with FOG would have an increased STV^VR^ when performing the VR task compared to non-freezers; *iii)* that there would be an increased STV^VR^ in the five steps preceding a freezing episode compared to five consecutive steps that were unrelated to freezing; and *iv)* that there would be a progressive shortening in the step latency in the three steps directly preceding a VR freezing episode.

## Methods

### Patient Details

A total of 28 patients with idiopathic PD were recruited for this study from the Parkinson’s Disease Research Clinic at the Brain and Mind Research Institute, University of Sydney. All patients satisfied UKPDS Brain Bank criteria and were deemed unlikely on MDS guidelines to have dementia [19] or major depression according to DSM-IV [20]. For reporting purposes we included the Mini Mental State Examination (MMSE) and Beck’s Depression Inventory version-II (BDI). Motor severity was assessed on the motor sub-score of the Unified Parkinson’s Disease Rating Scale (UPDRS-III) [21]. Finally, the Levodopa Equivalent Daily Dose (LEDD) was calculated for each patient [22].

Freezers were identified by self-reported freezing behaviour (positive response to item three of the Freezing of Gait Questionnaire: “Do you feel that your feet get glued to the floor while walking, making a turn or when trying to initiate walking (freezing)?”) [23] and clinically observed freezing behaviour by a neurologist (SJGL). The Freezing of Gait Questionnaire (FOG-Q) was included in the analyses for reporting purposes. Patients were assessed twice, once during their practically-defined ‘Off’ state following overnight withdrawal of anti**-**Parkinson medication and once whilst on their regular PD medication (defined as the ‘On’ state). The time between the two measurements was at least two weeks (average of nine weeks) and the order of assessment was randomized.

### Ethics Statement

This study was approved for by The University of Sydney Human Research and Ethics Committee and has therefore been performed in accordance with the ethical standards laid down in the 1964 Declaration of Helsinki. Written informed consent was obtained from all subjects.

### Virtual Reality Task

**Figure 1:**
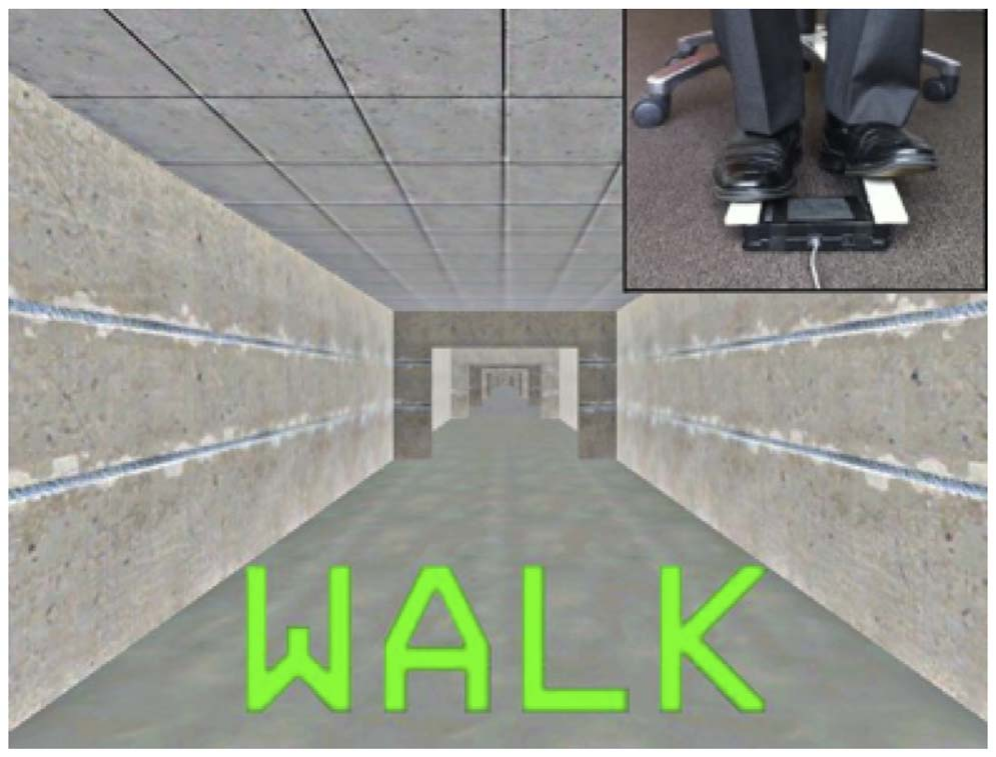
Representation of the virtual corridor in the Virtual Reality gait task including a wide ‘doorway’ and a ‘walk’ cue.

The VR task consisted of a three-dimensional virtual environment presented in the first-person on a computer screen. This was comprised of a straight corridor without any distracting cues except for wide ‘doorways’ (see Figure 1). Left and right foot pedals had to be depressed alternately ∼ 30 degrees below parallel to activate a trigger mechanism built into the foot pedals. This led to ‘physiological’ footstep sequences (i.e. left-right-left-right), which resulted in forward progression in the virtual environment. Patients were instructed to tap the pedals in a rhythm consistent with their normal gait (∼2 Hz) and to start tapping in response to a ‘WALK’ cue at the start of the task, and to cease this movement when a ‘STOP’ cue appeared at the end of the task. Both of these cues were presented at the bottom third of the screen in the colours green and red respectively. Further details are described in detail elsewhere [16], [17], however the major difference from the other published studies was that in this experiment patients were not presented any other cues, thus avoiding environmentally salient features (such as narrow doorways and turns) and any dual-task component. To familiarize the patients with the task, they performed a brief (∼1 minute) practice session before performing the experimental trial.

### Measures

The virtual reality program logged subject-independent time points (in seconds), such as the presentation of a simple WALK or STOP cue and the presence of a wide doorway. The steps associated with navigating a wide doorway were excluded from the analyses. The program also logged subject-dependent time points associated with the depression of a foot pedal. This allowed for the calculation of footstep latencies (time in seconds between two subsequent foot pedal depressions) associated with the successful completion of two steps (left-right), as well as any out-of-sequence footsteps (left-left or right-right), which were excluded from all analyses. For each subject the average footstep latencies of the left foot were compared to those of the right foot during both medication states. Step time variability in the VR was calculated as the step-to-step variability in footstep latencies by using the coefficient of variation (Coefficient of Variation = 100*Standard Deviation/Mean) [24]. The coefficient of variation is a measure that has been employed previously to quantify the temporal variability of human gait in several populations, such as older adults [25], elderly that experience frequent falls [26], patients with dementia [27], as well as in PD patients [6]–[9], [24].

Footstep latencies were also used to calculate each patient’s modal footstep latency. This was derived from an individual’s most frequent footstep latency whilst performing the VR gait task (as grouped into bins of 0.1 seconds). This footstep latency mode was taken as the most accurate measure of the cadence of natural stepping [15], [17], [18]. For each patient, footstep latencies greater than twice their modal footstep latency were defined as VR freezing episodes [15], [17], [18]. The percentage time spent with freezing (%FOG) was calculated from the time spent with VR freezing in relation to the total time spent performing the task. This gait metric has recently been proposed as a more reliable measure of freezing behaviour than simply recording the number of events [28].

To explore the STV^VR^ of the five steps preceding a VR freezing episode, a similar method was used as described in the study by Moreau et al [11]. That study showed that in freezers in their ‘Off’ state, the five steps preceding a freeze were characterized by an increased stride-length variability compared to five randomly selected steps that were unrelated to a freezing episodes [11]. Similarly, we used the STV^VR^ to compare the five steps preceding a VR freezing episode (pre-FOG) with an equal number of random steps that were not associated with a wide doorway or VR freezing episode (non-FOG) in freezers in their ‘Off’ state. Finally, the footstep latencies of the three steps directly preceding a VR freezing episode were explored for a possible sequence effect by looking at the step latency.

### Data Analysis

Data analysis was performed using IBM Statistical Package for the Social Sciences (SPSS) version 20. A Chi-square test was used for gender as it was categorized as a dichotomous variable. The other demographic group differences were analyzed with a multivariate analysis of variance (MANOVA), using the Pillai’s Trace as the overall significance test. The MMSE, BDI and FOG-Q scores were not included in the MANOVA due to a non-normal data distribution. Instead, a Mann-Whitney U test was used to compare the groups for these scores.

The STV^VR^ and %FOG data also violated the normality assumptions and therefore non-parametric tests were used. A Mann-Whitney U test was used to compare these measures between the groups and a Wilcoxon signed rank test was used to analyze the differences between the ‘On’ and ‘Off’ states within the groups and to compare the STV^VR^ of the five steps prior to a VR freeze (pre-FOG) with five consecutive steps that were not confounded by a freezing episode or other triggers (non-FOG). To ensure that there were no relationships between the STV^VR^ and the scores on the MMSE, a Spearman’s rho correlation analysis was performed.

Finally, a paired samples t-test was used to compare the modal footstep latencies within the groups between the ‘On’ and ‘Off’ states and to compare the average step times between the left- and the right foot.

The hypotheses were tested one-sided, as it was postulated *a priori* that both freezers and non-freezers would have a greater STV^VR^ with more VR freezing in their ‘Off’ state [7]–[10]; that freezers would show a greater STV^VR^ and have more VR freezing compared to non-freezers [8]; that the pre-FOG steps would have greater STV^VR^ than the non-FOG steps [11]; and that the footstep latencies of the three steps prior to a VR freezing episode would show a sequence effect related to latency [13]. Alpha levels were set at 0.05.

## Results

### Demographic Statistics

The demographics are presented in Table 1. The groups (17 freezers and 11 non-freezers) were matched for age, disease duration, Hoehn and Yahr stages, LEDD, UPDRS-III ‘Off’ and ‘On’ medication state assessments and modal footstep latencies in both the ‘Off’ and ‘On’ state (F (8,19) = 1.089, p = 0.412). The non-significant difference between the UPDRS-III ‘Off’ states was further reduced when excluding item 29 regarding gait (Freezers: Mean (SD) = 28.2 (12); Non-freezers: Mean (SD) = 20.7 (10), t (26) = 1.67, p = 0.106). Fifteen freezers were male (Chi^2^ (1) = 9.94, p<0.01) and eight non-freezers were male (Chi^2^ (1) = 2.27, p = 0.132). Freezers scored significantly higher on the FOG-Q (U = 3.50, Z = −4.25, p<0.001), had similar BDI scores (U = 56.5, Z = −1.74, p = 0.081), but scored significantly lower on the MMSE (U = 52.0, Z = −2.02, p = 0.044) compared to non-freezers.

**Table 1:** Demographic, neurological and freezing characteristics of subjects with and without freezing of gait.

		Freezers (n = 17)				Non-freezers (n = 11)		
		Mean ± SD	Median	Range		Mean ± SD	Median	Range
Age		66.1**±**6.5	66		25	63.4**±**8.1	64	30
Disease duration (yr.)		7.24**±**3.7	7.0		25	5.55**±**3.1	4.0	11
LEDD		807**±**549	800		2400	517**±**427	450	1350
H & Y		1.88**±**0.4	2.0		1.5	1.77**±**0.6	2.0	1.5
UPDRS III	‘Off’	32.4**±**11	36		40	22.2**±**11	24	38
UPDRS III	‘On’	24.2**±**14	22		44	12.5**±**6.7	11	19
MMSE		27.8**±**2.1	28		7.0	29.3**±**0.6	29	2.0
BDI-II		13.9**±**11	14		42	7.45**±**6.5	4.0	18
Modal FSL	‘Off’	0.42**±**0.2	0.37		0.55	0.47**±**0.2	0.44	0.57
Modal FSL	‘On’	0.46**±**0.1	0.49		0.50	0.46**±**0.1	0.44	0.44
FOG-Q		10.2**±**4.5	10		15	1.30**±**1.4	1.0	4.0

NOTE: H & Y = Hoehn and Yahr stages, UPDRS III = motor section of the Unified Parkinson’s Disease Rating Scale, LEDD = Levodopa Equivalent Daily Dose (mg/day), MMSE = Mini Mental State Examination, BDI-II = Beck’s Depression Inventory version II, Modal FSL = Modal Footstep Latency, FOG-Q = Freezing of Gait Questionnaire.

The modal footstep latencies were similar between the ‘Off’ and ‘On’ states for both groups (freezers: t (26) = −1.97, p = 0.067; non-freezers: t (10) = 0.141, p = 0.891). Finally, the average step times between the left- and right foot were the same for both groups in both the medication states (freezers ‘Off’: t (16) = 1.825, p = 0.089; freezers ‘On’: t (16) = 1.462, p = 0.163; non-freezers ‘Off’: t (10) = 1.301, p = 0.222; non-freezers ‘On’: t (10) = 1.264, p = 0.235).

### Virtual Reality Freezing

Freezers had more percentage time frozen (%FOG) than non-freezers in the ‘Off’ state (U (27) = 58.5, Z = −1.65, p = 0.049), while no group difference was found in the ‘On’ state (U (27) = 83, Z = −0.50, p = 0.309). Freezers showed a higher %FOG in their ‘Off’ state compared to their ‘On’ state (Z (16) = −1.76, p = 0.039). This difference was not found between the medication states for non-freezers (Z (11) = −0.059, p = 0.477).

### Step Time Variability in the VR

The results of the step time variability analyses are presented in Table 2. Each subject took more than 280 steps during each VR trial (Mean (SD) = 560 (6.2)). A greater STV^VR^ was confirmed in the ‘Off’ state compared to the ‘On’ state for both freezers and non-freezers. Moreover, freezers showed a significantly greater STV^VR^ compared to non-freezers in their ‘Off’ state. Freezers also showed greater STV^VR^ in the ‘On’ state however, this result did not reach statistical significance. Finally, no significant correlations were found between the STV^VR^ and the MMSE scores for both freezers and non-freezers in their ‘Off’ and ‘On’ states (freezers ‘Off’: r = −.103, Freezers ‘On’: r = −.169, non-freezers ‘Off’: r = −.087, non-freezers ‘On’: r = −.056, all p-values are non-significant).

**Table 2:** Differences in Step Time Variability in the Virtual Reality gait task (STV^VR^) between Freezers and Non-freezers and between the ‘Off’ and ‘On’ states.

		STV^VR^ Median - Range	STV^VR^ Median - Range	p-value
**Group effect**		*Freezers (n = 17)*	*Non-freezers (n = 11)*	
	*‘Off’*	24.5–69.8	15.6–19.7	p = 0.032
	*‘On’*	13.5–48.4	11.8–15.7	p = 0.125
**Medication**		*‘Off’*	*‘On’*	
	*Freezers (n = 17)*	24.5–69.8	13.5–48.4	p = 0.018
	*Non-freezers (n = 11)*	15.6–19.7	11.8–15.7	p = 0.017

NOTE: Medication = differences between STV^VR^ in the *‘*Off’ and ‘On*’* medication states.

The three steps prior to a VR freezing episode in freezers ‘Off’ their medication were not characterized by a progressive reduction in footstep latencies, with mean footstep latencies of: 0.998, 1.213 and 1.015 seconds for the steps 3, 2 and 1 before a VR freezing episode, respectively. However, a greater STV^VR^ was confirmed in freezers ‘Off’ their medication when comparing the five steps directly preceding a VR freeze with five randomly selected steps that were unrelated to a VR freezing episodes (t (9) = 2.56, p = 0.016).

## Discussion

The aim of the current study was to determine whether a VR task could reproduce the key features of gait variability associated with FOG in PD [6]–[8], [11]. Analysis of STV^VR^ revealed major similarities to those reported for stride time variability in previous studies of patients performing actual gait tasks. Specifically, the STV^VR^ was greater in freezers compared to non-freezers and was partially ameliorated by dopaminergic therapy. Additionally, there was an increased STV^VR^ just prior to a freezing episode, but we were unable to demonstrate a sequence effect on latency across the three steps directly preceding a freeze.

Both freezers and non-freezers showed increased variability in their motor rhythm when withdrawn from dopaminergic medication. These results are consistent with previous work that has shown that PD patients have a greater stride time variability in their ‘Off’ state during a 6 m walking task in the home environment [9]. In addition, Hausdorff *et al* [7] showed that the stride time variability was reduced in response to L-dopa for both freezers and non-freezers by using an 80 metre walking course and force-sensitive insoles.

In the VR environment, freezers displayed greater temporal variability in their stepping rhythm compared to non-freezers in the ‘Off’ state, whilst the modal footstep latency did not differ between the groups. These results mirror the findings of studies showing that the stride CV is almost twice as high amongst freezers compared to non-freezers, whilst the average stride times do not differ between these patient groups [7], [8].

The STV^VR^ of the five steps preceding a virtual freezing episode was explored by using a method based on the study by Moreau *et al* [11]. That study assessed ten patients with FOG while they walked down a 7-m walkway at high velocity while ‘Off’ their regular medication. After determining the timing of the freezing episodes, the investigators then compared the five pre-FOG steps with five randomly chosen steps that were not associated with a freezing episode (non-FOG). They found that the five steps leading up to a freezing episode (pre-FOG) were characterized by increased variability in length [11]. The current study showed a similar pattern of results when comparing the STV^VR^ of the pre-FOG steps to the STV^VR^ of the non-FOG steps in freezers ‘Off’ their medication.

Whilst the current study failed to demonstrate a shortening in the footstep latencies of the three steps directly preceding a VR freezing episode, it should be noted that this sequence effect has previously been reported in the context of reducing stride length rather than step timing. Future studies would be needed to investigate whether a sequence effect is present in the amplitude of the foot movements during the performance of the VR gait task.

Previous studies using the VR task [14], [16], [17] have included an additional ‘dual-task performance’ along with a series of salient environmental features (such as narrow doorways [14], both of which are known to provoke freezing behaviour [29], [30]. Instead, the current study utilised only simple WALK and STOP cues, removing the possibility of the influence of these added features. However, despite this less challenging VR task, the present study showed that freezers still had greater %FOG compared to non-freezers in their ‘Off’ state and that freezers froze more in the ‘Off’ state compared to their ‘On’ state. These results highlight the role of dopamine in the partial amelioration of freezing behaviour by taking Levodopa, however it is not clear from the current study whether such a positive benefit would be conferred onto patients with ‘on’ freezing [31]. Despite this reservation, these results are consistent with a large body of clinical research that shows that FOG is exacerbated by low levels of dopaminergic medication [7]–[10], and together with the results of previous work [32], even supports the notion that the VR paradigm is capable of detecting freezing episodes. However, we acknowledge that future research should also combine the VR task with additional techniques, such as accelerometry to determine whether the VR freezing observed manifest features observed in FOG like ‘trembling in place’ [33].

The current study did not record actual gait parameters from the subjects who were assessed with the VR gait task. This approach would enhance the interpretability of the results presented and will form the basis of future work. This study also analyzed step time variability instead of stride time variability, effectively including asymmetry in the measure of the within-person standard deviation. Therefore the ‘gait asymmetry’ seen in this study might have been concordant with the dominant symptom side of the patients. However, analysis showed that there were no differences in the average step times between the left- and right foot for both freezers and non-freezers in both medication states. This indicates that the results found in this study were not due to an increased step time on the symptom dominant side of the patients.

Moreover, to determine gait disturbances and to control for differences in individual patient’s footstep latencies, all measures were scaled to each patient’s modal footstep latency. These modal footstep latencies were the same between freezers and non-freezers and between the ‘Off’ and ‘On’ states for both groups. The groups were also matched for the UPDRS-III scores, although non-freezers displayed a slight bias towards less severe motoric disease symptoms. Thus, the significant group differences reported were unlikely to be related to general motor dysfunction and appear to relate more specifically to some shared pathophysiology underlying the phenomenon of FOG.

In conclusion, the VR gait task appears able to reproduce the key features of stride time variability allowing for a model of freezing of gait to be explored. Additional research is now needed to complete this validation and it is hoped that this novel approach may help in the understanding of the pathophysiology underlying FOG.
